# Hepatocyte growth factor stimulates neutrophil degranulation but not respiratory burst

**DOI:** 10.1155/S0962935193000195

**Published:** 1993

**Authors:** I. C. Kowanko, A. Ferrante, T. Nakamura

**Affiliations:** 1 Departments of Immunology and University Department of Paediatrics Women's and Children's Hospital North Adelaide Australia; 2 Department of Biology Kyushu University Fukuoka Japan

## Abstract

Neutrophil function is regulated in part by cytokines with growth factor activities for different cell types. Hepatocyte growth factor (HGF) is a cytokine produced during injury to the liver and other organs. Neutrophils are numerous in such tissue injury sites and may be influenced by HGF. In the present study the effect of HGF on neutrophils was investigated. The data show that HGF at 1–10 ng/ml increased lysosomal enzyme release from both specific and azurophilic granules of cytochalasin-B treated neutrophils. The release of specific granule contents in response to N-formyl-methionyl-leucylphenylalanine was also increased by HGF. In contrast there were no significant effects of HGF on neutrophil respiratory burst, adherence or locomotion. It is concluded that HGF modulates neutrophil granule exocytosis.


Mediators of Inflammation, 2, 129–133 (1993)

### Hepatocyte growth factor stimulates neutrophil degranulation but not respiratory burst

I. C. Kowanko,[1,CA] A. Ferrante[1] and T. Nakamura[2]

[1]Departments of Immunology and University Department of Paediatrics, Women's and Children's Hospital, North Adelaide, Australia; [2]Department of Biology, Kyushu University, Fukuoka, Japan

[CA] Corresponding Author

**NEUTROPHIL function is regulated in part by cytokines with growth factor activities for different cell types. Hepatocyte growth factor (HGF) is a cytokine produced during injury to the liver and other organs. Neutrophils are numerous in such tissue injury sites and may be influenced by HGF. In the present study the effect of HGF on neutrophils was investigated. The data show that HGF at 1–10 ng/ml increased lysosomal enzyme release from both specific and azurophilic granules of cytochalasin-B treated neutrophils. The release of specific granule contents in response to *N*-formyl-methionyl-leucyl-phenylalanine was also increased by HGF. In contrast there were no significant effects of HGF on neutrophil respiratory burst, adherence or locomotion. It is concluded that HGF modulates neutrophil granule exocytosis.**

**Key words:** Adherence, β-Glucuronidase, Chemiluminescence, Cytokine, Locomotion, Lysosomal enzyme, Polymorphonuclear leukocyte, Superoxide, Vitamin $B_{12}$ binding protein

### Introduction

Hepatocyte growth factor (HGF) is a potent mitogen for hepatocytes and is found in the sera of animals with liver injuries.[1] It is now known that HGF is a pleiotropic factor (reviewed in References 2 and 3) with effects including promotion of epithelial cell growth,[2] inhibition of proliferation of several tumour cell lines,[4] enhancement of epithelial cell motility and identity with scatter factor[5] and induction of tubule formation by epithelial cells.[2] The cDNA of HGF has been cloned and the HGF molecule has been characterized as a heterodimer consisting of a 65 kDa α chain and a 34 kDa β chain, structurally similar to plasmin.[3] HGF is produced by Kupffer cells in the liver, and also in other organs such as lung and kidney, not only in liver injury but also after renal damage (reviewed in References 2 and 3). The HGF receptor is the c-*met* proto-oncogene product, and is widely distributed.[6]

Neutrophil granulocytes are important mediators of both host defence against microbial attack and tissue damage by virtue of their armament of lysosomal granule enzymes and products of the respiratory burst. The function of mature neutrophils can be regulated by cytokines and growth factors such as interleukin-1 (IL-1),[7,8] tumour necrosis factor-alpha (TNFα),[7,9] and granulocyte macrophage colony-stimulating factor (GM-CSF)[10,11]. HGF, like IL-1 and GM-CSF, can be produced by cells of the monocyte lineage.[12,13] Neutrophil counts are likely to be elevated during the traumatic injuries which can lead to high HGF production. A form of HGF known as tumour cytotoxic factor derived from fibroblasts induces differentiation of HL-60 cells into the mature granulocyte phenotype.[14] GM-CSF, which promotes the differentiation of granulocyte precursors also enhances the function of mature granulocytes.[10,11,15] Therefore HGF was examined for its effects on neutrophil functions.

### Materials and Methods

*Hepatocyte growth factor:* Human recombinant HGF was produced in transformed Chinese hamster ovary cells.[16] A sterile stock solution of HGF at 200 ng/ml in Hank's balanced salt solution (HBSS) containing 0.2% bovine serum albumin (BSA; Commonwealth Serum Laboratories, Melbourne, Australia) was stored in aliquots at −80°C. Aliquots were thawed once only, and diluted in HBSS with 0.2% BSA as required for each experiment. The diluent was sterilizsed by filtration (0.2 μM) and contained <0.125 endotoxin units/ml by *Limulus* lysate assay.

*Preparation of neutrophils:* Human neutrophils were isolated from heparinized peripheral blood from healthy adult volunteers by centrifuging through Ficoll–Hypaque (density = 1.114 g/l) for 30 min.[17] The neutrophils were 98% pure, >99% viable as judged by trypan blue exclusion, and were used within 30 min.

*Measurements of neutrophil degranulation:* β-glucuronidase and vitamin $B_{12}$ binding protein (Vit $B_{12}$ BP),

© 1993 Rapid Communications of Oxford Ltd markers of azurophilic and specific granules, respectively, were measured as described previously.[10] Briefly, $10^6$ neutrophils were preincubated for 20 min (unless otherwise stated) at 37°C with or without HGF (0.1–10 ng/ml), and then with or without cytochalasin-B (2 $\mu$g/ml final, Sigma) for 10 min. The cells were then stimulated for 30 min with N-formyl-methionyl-leucyl-phenylalanine (FMLP, Sigma, $5 \times 10^{-6}$ M final), or HBSS control, the volume adjusted to 1 ml with HBSS and supernatants analysed for enzyme content. In some experiments phorbol myristate acetate (PMA, $10^{-9}$ or $10^{-8}$ M) or tumour necrosis factor-alpha (TNF$\alpha$, 100 U/ml) were used as stimuli. $\beta$-glucuronidase activity was determined by a modified fluorometric method[7,18] and Vit $B_{12}$ BP was measured using a radiometric charcoal binding assay.[19] Results were expressed as a percentage of total cell content (Triton disrupted cells). Neutrophil viability was measured as the release of the cytoplasmic enzyme lactate dehydrogenase (LDH) as described previously.[20]

*Measurement of neutrophil respiratory burst:* Neutrophil chemiluminescence was measured essentially as described previously[10] using conditions for preincubation with HGF/cytochalasin-B as for degranulation assays. The stimuli (FMLP, etc.) or HBSS control and lucigenin (Sigma, 250 $\mu$M final) were added to the tubes immediately before placing them into the luminometer (Model 1250, LKB Wallac, Turku, Finland). Maximal rate of chemiluminescence was recorded as mV. Superoxide dismutase inhibitable superoxide production was measured as described previously[10] after preincubation of neutrophils with HGF as above, and stimulation with or without FMLP.

*Adherence of neutrophils:* Neutrophil adherence to plasma coated plastic microtitre wells was measured as described[21] using a modified spectrophotometric assay.[22]

*Expression of results and statistics:* Within each experiment, assays were conducted in at least triplicate, using neutrophils from a single donor. Each experiment was conducted three times or more with neutrophils from different donors. Results are presented as means (±S.E.M. or range) of replicate experiments. The significance of the data was tested using Wilcoxon's matched pairs signed ranks test.

### Results

*Effects of HGF on neutrophil granule exocytosis:* HGF induced the degranulation of neutrophils. Preincubation with recombinant human HGF for 20 min increased the release of both specific and azurophilic granule components from cytochalasin-B treated neutrophils in a dose dependent manner (Fig. 1). HGF concentrations during preincubation of 1, 5 and 10 ng/ml enhanced the release of Vit $B_{12}$ BP, a marker of neutrophil specific granules, to almost double the release from control neutrophils (Table 1, Fig. 1). The release of $\beta$-glucuronidase (azurophilic granule marker) was also augmented by HGF at 5 and 10 ng/ml (Table 1, Fig. 1). Although these increases were relatively small (granule release was less than 10% of the total cell content, even with HGF stimulus), they were statistically significant in a group of nine separate experiments performed with neutrophils from different donors, evaluated with Wilcoxon's matched pairs signed ranks test (Fig. 1). The effects of HGF were observed with incubation times varying from 10 min to 2 h (results not shown).

The release of Vit $B_{12}$ BP from cytochalasin-B treated neutrophils in response to FMLP was also

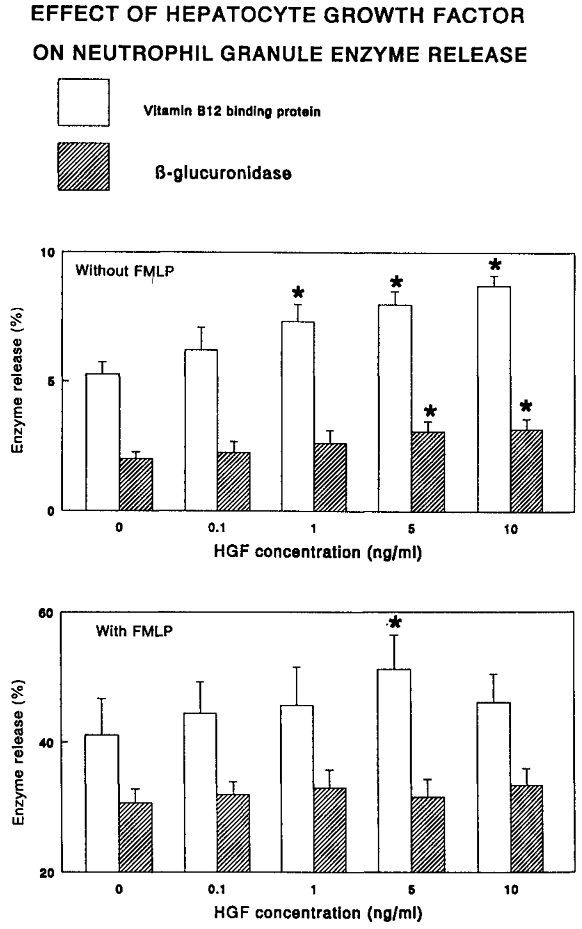

FIG. 1. Effect of hepatocyte growth factor on neutrophil granule enzyme release. Neutrophils were preincubated for 20 min with the indicated concentrations of HGF, followed by 10 min with cytochalasin-B and then 30 min with $5 \times 10^{-6}$ M FMLP (lower graph) or without FMLP (upper graph). The release of vitamin $B_{12}$ binding protein (open bars) and $\beta$-glucuronidase (hatched bars) was measured and is expressed as percentage of total cell content. The mean and S.E.M. of nine experiments is shown. *, indicates $p < 0.05$ for the effect of HGF compared to control using Wilcoxon's paired test.

**Table 1.** Effect of HGF on neutrophil degranulation, respiratory burst and adherence[a]

| Assay | Stimuli[b] | HGF[d] Percent of control value Mean | (range) | Control Assay value[c] | Number of expts |
|---|---|---|---|---|---|
| Vit $B_{12}$BP | cytoB | 180 | (112–260) | 5.3% release | 9 |
| | cytoB + FMLP | 120 | (88–184) | 41.1% | 8 |
| | Diluent | 113 | (77–160) | 9.6% | 4 |
| | FMLP | 112 | (101–133) | 11.5% | 3 |
| $\beta$-Glucuronidase | cytoB | 160 | (95–276) | 2.0% | 8 |
| | cytoB + FMLP | 103 | (72–118) | 30.6% | 7 |
| | Diluent | 144 | (82–229) | 3.9% | 3 |
| | FMLP | 119 | (100–157) | 4.6% | 3 |
| Chemiluminescence | cytoB | 115 | (56–198) | 2.1 mV | 11 |
| | cytoB + FMLP | 106 | (62–171) | 159.6 mV | 12 |
| Adherence | Diluent | 136 | (32–276) | 0.22 ΔOD | 9 |

[a] Neutrophils were preincubated with either HGF (10 ng/ml) or diluent control for 20 min before the addition of stimuli. [b] Neutrophils were further treated with various combinations of cytochalasin-B (2 μg/ml final) or diluent for 10 min, followed by FMLP ($5 \times 10^{-8}$M final) or diluent. [c] Assay values for control neutrophils (without HGF) are shown as mean percent of total cell content released for Vit $B_{12}$ BP and $\beta$-glucuronidase, maximal rate of light emission in mV for chemiluminescence, and change in optical density at 570 nm for adherence. [d] The effect of HGF is expressed as a percentage of the control assay value (mean and range) for the specified number of experiments.

potentiated by HGF at 5 ng/ml (Fig. 1). Other concentrations of HGF did not significantly enhance FMLP elicited Vit $B_{12}$ BP release, nor was $\beta$-glucuronidase release significantly increased after these conditions (Fig. 1, Table 1). HGF did not affect neutrophil granule enzyme release in response to PMA or TNFα (results not shown).

LDH release from neutrophils was not detectable without FMLP stimulation and was always <7% with FMLP. LDH release was unaffected by HGF (results not shown), indicating that HGF did not impair cell viability or cytosol membrane integrity.

In experiments in which cytochalasin-B was omitted there was no significant effect on HGF on either Vit $B_{12}$ BP or $\beta$-glucuronidase release, nor was there any consistent or significant effect of HGF on FMLP elicited degranulation under these conditions (Table 1).

Treatment of neutrophils with 100 U/ml of TNF for 30 min is known to prime the cells for increased enzyme release,[7] and HGF was examined for its ability to modify this function. Incubation of neutrophils with 10 ng/ml HGF immediately after TNF treatment had no consistent effect on release of either Vit $B_{12}$ BP or $\beta$-glucuronidase, with or without additional FMLP stimulation (results not shown).

*Effect of HGF on neutrophil respiratory burst:* Lucigenin enhanced neutrophil chemiluminescence was used to measure the respiratory burst. Neutrophils were preincubated for 20 min with a range of concentrations of HGF, followed by 10 min with cytochalasin-B, before monitoring luminescence in response to FMLP or diluent. HGF at 1 and 10 ng/ml during preincubation increased basal chemiluminescence slightly (Table 1) but this increase was not statistically significant (Wilcoxon's paired test, eleven experiments). FMLP induced chemiluminescence was not significantly altered by HGF (Table 1). Increasing HGF concentration to 100 ng/ml during preincubation did not affect either basal or FMLP stimulated neutrophil chemiluminescence (results not shown). There was no effect of varying the time of preincubation with HGF; times ranging from 10 min up to 2 h were tested (results not shown).

In the absence of cytochalasin-B there were no consistent effects of HGF on neutrophil chemiluminescence (results not shown). Chemiluminescence of TNF primed neutrophils was unaffected by HGF at concentrations up to 10 ng/ml (four experiments, results not shown).

Superoxide release from neutrophils was also measured as superoxide dismutase inhibitable cytochrome-C reduction. Preincubation of cells for 20 min with 10 ng/ml HGF had no consistent effects either on basal or FMLP elicited superoxide responses (97% and 104% of controls without HGF, respectively).

*Effect of HGF on neutrophil adherence:* Neutrophil adhesion to plasma-coated plastic in the presence of a range of concentrations of HGF was examined in nine separate experiments using neutrophils from different donors. There was no consistent effect on HGF on adherence (Table 1) and responses varied widely between experiments, with no apparent dose–response relationship over the concentration range 0.01–10 ng/ml (results not shown).

*Effects of HGF on neutrophil locomotion:* Pretreatment of neutrophils with 0.01–10 ng/ml of HGF did not alter their random migration under agarose, nor their migration towards the chemotactic agent FMLP (results not shown).

### Discussion

It is reported here that recombinant human HGF induces the release of granule enzymes from neutrophils. Secretion of both Vit $B_{12}$ BP and $\beta$-glucuronidase, markers of the specific and azurophilic granules, respectively, was enhanced up to 200%, suggesting that HGF is a complete secretagogue for human neutrophils. The effect was dose dependent and required only 10 min pre-incubation. HGF shares this ability to induce the exocytosis of both types of neutrophil granule with other cytokines e.g. IL-1[7,8] and TNF$\beta$.[23] The magnitude of the increase in enzyme release caused by HGF was similar to that observed with TNF$\beta$,[23] GM-CSF[10] and interferon gamma (IFN-$\gamma$),[24] stimulation of neutrophils. The increased release of granule enzymes from HGF treated neutrophils was not due to HGF toxicity because viability (LDH release) was not altered.

Neutrophil specific granule release (Vitamin $B_{12}$ BP) in response to FMLP/cytochalasin-B was increased by HGF at 10 ng/ml only. There was no effect on $\beta$-glucuronidase release under these conditions, nor was there any effect of HGF on TNF priming of neutrophil exocytosis. These results suggest that HGF does not prime neutrophils for an increased response to subsequent stimulation in contrast to other cytokines such as TNF$\alpha$,[7,9] IFN-$\gamma$[24,25] or GM-CSF.[10,11] Cytochalasin-B was required in these experiments in order for HGF to exert a measurable effect on basal enzyme release. Cytochalasin-B is a fungal metabolite which interferes with the microtubule contractile system and phagocytosis.[26] It enhances granule release and respiratory burst in response to otherwise weak or inactive soluble or particulate agonists.[26] The release of neutrophil lysosomal enzymes in response to IL-1 and TNF$\alpha$ also required cytochalasin-B.[7,8]

The present data show that neutrophil respiratory burst activity was not significantly altered by treatment with 0.1–100 ng/ml HGF compared to diluent. These results apparently contrast with a recent report that HGF primes neutrophils for increased luminol enhanced chemiluminescence.[27] We measured lucigenin enhanced chemiluminescence which is a measure of superoxide release,[28] the primary oxygen radical produced by neutrophils. Since chemiluminescence enhanced by luminol measures formation of other down-stream reactive oxygen metabolites such as hypochlorous acid which is dependent on myeloperoxidase release from the specific granules,[28] the results of Jiang *et al.*[27] may reflect an increased release of myeloperoxidase rather than increased respiratory burst activity.

It is unlikely that the increase in granule release seen with HGF was due to contamination with bacterial endotoxin because there was no effect on respiratory burst which would be expected with lipopolysaccharide.[28] The recombinant HGF was prepared in transformed mammalian cells and the stock solution (200 ng/ml) did not contain any measurable TNF$\beta$, TNF$\alpha$ or IFN-$\gamma$ (i.e. $<100$ pg/ml) by enzyme-linked immunosorbent assay performed in our laboratory. There was also no effect of HGF on the other neutrophil functions that were tested. These included adherence to plasma-coated plastic and random or chemotactically directed migration under agarose. Taken together these results suggest that HGF is able to induce neutrophil granule release without affecting other functions.

It is not known if neutrophils express receptors for HGF, but this receptor, known to be the c-*met* proto-oncogene product, is widely distributed on many cell types.[6] The high affinity receptor on hepatocytes has a $K_d$ of 60–90 pM, corresponding to about 5–8 ng/ml.[2] This study and others which describe effects of HGF on neutrophils[27] and the myeloid cell line HL60[14] suggest that neutrophils might bear HGF receptors.

Other growth factors can modulate neutrophil activities. For example growth hormone and insulin-like growth factor-1 prime neutrophils to secrete superoxide.[29] Platelet derived growth factor upregulates the 5-lipoxygenase pathway in differentiating neutrophils.[30] Transforming growth factor-beta induces precursor cells (CFU-GM) to differentiate preferentially into granulocytes.[31] Other cytokines which influence cell growth and differentiation such as the interleukins and colony-stimulating factors, can activate neutrophil functions including adhesion, migration inhibition, antibody dependent cytotoxicity, triggering of respiratory burst and degranulation, priming for increased responses to stimuli, microbicidal action and cartilage damage.[7–11,21–25]

Since HGF affects differentiation of granulocytes[14] these findings support the hypothesis that factors which regulate myeloid growth and differentiation also modulate the function of the mature cell. HGF is produced in several conditions such as hepatic failure and renal injury.[1–3] Neutrophils are likely to encounter high concentrations of HGF at such times. For example serum HGF concentrations of $>1$ ng/ml have been reported in 82% of patients with fulminant hepatic failure, rising to 5–10 ng/ml immediately after plasma exchange.[32] HGF concentrations in the microenvironment around the HGF-producing cells at injury sites are probably even higher.

Neutrophil precursor cells have also been reported to produce HGF.[12] It has already been shown that HGF is a pleiotropic factor with endocrine, paracrine and autocrine effects on a number of different cell types.[1–3] In this paper it is reported that HGF at physiological concentrations induces lysosomal enzyme release from neutrophils. Since neutrophils are so numerous and have a half-life in the circulation of only about 7 h, their combined enzyme release in response to HGF could be significant. Other cytokines, such as TNF$\beta$, GM-CSF, TNF$\alpha$ and IFN-$\gamma$, which increase neutrophil azurophilic granule release can also augment neutrophil mediated cartilage damage *in vitro*.[21,23] Such host tissue damage is in large part due to lysosomal enzymes such as elastase.[33] HGF is likely to be one of many growth factors and cytokines which contribute to the complex regulation of immune cell functions.

### References

1. Nakamura T, Nawa K, Ichihara A. Partial purification and characterization of hepatocyte growth factor from serum of hepatectomized rats. *Biochem Biophys Res Commun* 1984; **122:** 1450–1459.
2. Matsumoto K, Nakamura T. Hepatocyte growth factor: molecular structure, roles in liver regeneration, and other biological functions. *Crit Rev Oncogen* 1992; **3:** 27–54.
3. Nakamura T. Structure and function of hepatocyte growth factor. *Prog Growth Factor Res* 1991; **3:** 67–85.
4. Tajima H, Natsumoto K, Nakamura T. Hepatocyte growth factor has potent antiproliferative activity in various tumor cell lines. *FEBS Lett* 1991; **291:** 229–232
5. Weidner KM, Behrens J, Vandekerchhove J, Birchmeier W. Scatter factor: Molecular characteristics and effect on invasiveness of epithelial cells. *J Cell Biol* 1990; **111:** 2097–2108.
6. Bottaro DP, Rubin JS, Faletto DL, *et al.* Identification of the hepatocyte growth factor receptor as the c-*met* proto-oncogene product. *Science* 1991; **251:** 802–804.
7. Ferrante A, Nandoskar M, Walz A, Goh DHB, Kowanko IC. Effects of tumour necrosis factor alpha and interleukin-1 alpha and beta on human neutrophil migration, resepiratory burst and degranulation. *Int Arch Allergy Appl Immunol* 1988; **86:** 82–91.
8. Smith RJ, Speziale SC, Bowman BJ. Properties of interleukin-1 as a complete segretagogue for human neutrophils. *Biochem Biophys Res Commun* 1985; **130:** 1233–1240.
9. Klebanoff SJ, Vadas MA, Harlan JM, *et al.* Stimulation of neutrophils by tumor necrosis factor. *J Immunol* 1986; **136:** 4220–4225.
10. Kowanko IC, Ferrante A, Harvey DP, Carman KL. Granulocyte-macrophage colony-stimulating factor augments neutrophil killing of *Torulopsis glabrata* and stimulates neutrophil respiratory burst and degranulation. *Clin Exp Immunol* 1991; **83:** 225–230.
11. Gasson JC, Weisbart RH, Kaufman SE, *et al.* Purified human granulocyte-macrophage colony-stimulating factor: direct action on neutrophils. *Science* 1984; **226:** 1339–1342.
12. Nishino T, Kaise N, Sindo Y, *et al.* Promyelocytic leukemia cell line, HL-60, produces hepatocyte growth factor. *Biochem Biophys Res Commun* 1991; **181:** 323–330.
13. Seki T, Ihara I, Sugimura A, *et al.* Isolation and expression of cDNA for different forms of hepatocyte growth factor from human leukocyte. *Biochem Biophys Res Commun* 1990; **172:** 321–327.
14. Shima N, Itagaki Y, Nagao M, Yasuda H, Morinaga T, Higashio K. A fibroblast-derived tumor cytotoxic factor/F-TCF (hepatocyte growth factor/HGF) has multiple functions *in vitro*. *Cell Biol Int Rep* 1991; **15:** 397–408.
15. Metcalf D. Molecular control of granulocyte and macrophage production. *Prog Clin Biol Res* 1985; **191:** 323–327.
16. Nakamura T, Nishizawa T, Hagiya M, *et al.* Molecular cloning and expression of human hepatocyte growth factor. *Nature* 1989; **342:** 440–443.
17. Ferrante A, Thong YH. Separation of mononuclear cells from human blood by the one-step Hypaque–Ficoll method is dependent on blood column height. *J Immunol Methods* 1982; **48:** 81–85.
18. Kolodny EJ, Mumford RA. Human leukocyte acid hydrolases: characterization of eleven lysosomal enzymes and study of reaction conditions for their automated analysis. *Clin Chim Acta* 1976; **70:** 247–257.
19. Gottlieb C, Lau KS, WAsserman LR, Herbert V. Rapid charcoal binding assay for intrinsic factor (IF), gastric juice unsaturated $B_{12}$ binding capacity, antibody to IF and serum unsaturated B12 binding capacity. *Blood* 1965; **25:** 875–883.
20. Bates EJ, Johnson CC, Lowther DA. Inhibition of proteoglycan synthesis by hydrogen peroxide in cultured bovine articular cartilage. *Biochim Biophys Acta* 1985; **838:** 221–228.
21. Kowanko IC, Ferrante A. Granulocyte-macrophage colony-stimulating factor augments neutrophil-mediated cartilage degradation and adherence. *Arthritis Rheum* 1991; **34:** 1452–1460.
22. Gamble JR, Vadas MA. A new assay for the measurement of the attachment of neutrophils and other cell types to endothelial cells. *J Immunol Methods* 1988; **109:** 175–184.
23. Ferrante A, Nandoskar M, Walz A, Goh DHB, Beard LJ. Tumour necrosis factor beta (lymphotoxin) inhibits locomotion and stimulates the respiratory burst and degranulation of neutrophils. *Immunology* 1988; **63:** 507–512.
24. Kowanko IC, Ferrante A. Stimulation of neutrophil respiratory burst and lysosomal enzyme release by human interferon-gamma. *Immunology* 1987; **62:** 149–151.
25. Cassatella MA, Cappelli R, DellaBianca V, Grzeskowiak M, Dusi S, Berton G. Interferon-gamma activates human neutrophil oxygen metabolism and exocytosis. *Immunol* 1988; **63:** 499–506.
26. Zurier RB, Hoffstein S, Weissmann G. Cytochalasin B: effect on lysosomal enzyme release from human leucocytes. *Proc Natl Acad Sci USA* 1973; **70:** 844–848.
27. Jiang W, Puntis MCA, Nakamura T, Hallett MB. Neutrophil priming by hepatocyte growth factor, a novel cytokine. *Immunol* 1992; **77:** 147–149.
28. Klebanoff SJ. Phagocytic cells: Products of oxygen metabolism. In: Gallin J, Goldstein IM, Snyderman R, eds. *Inflammation: Basic principles and Clinical Correlates.* New York: Raven Press, 1988; 391–444.
29. Fu Y-K, Arkins S, Wang BS, Kelley KW. A novel role of growth hormone and insulin-like growth factor-1. Priming neutrophils for superoxide anion secretion. *J Immunol* 1991; **146:** 1602–1608.
30. Goerig M, Habenicht AJR, Zeh W, *et al.* Evidence for co-ordinate, selective regulation of eicosanoid synthesis in platelet-derived growth factor-stimulated 3T3 fibroblasts and in HL-60 cells induced to differentiate into macrophages or neutrophils. *J Biol Chem* 1988; **263:** 19384–19391.
31. Chenu C, Pfeilschifter J, Mundy GR, Roodman GD. Transforming growth factor beta inhibits formation of osteoclast-like cells in longterm human marrow cultures. *Proc Natl Acad Sci USA* 1988; **85:** 5683–5687.
32. Tomiya T, Nagoshi S, Fujiwara K. Significance of serum human hepatocyte growth factor levels in patients with hepatic failure. *Hepatology* 1992; **15:** 1–4.
33. Ferrante A, Kowanko IC, Bates EJ. Mechanisms of host tissue damage by cytokine-activated neutrophils. In: Coffey RG, ed. *Granulocyte responses to cytokines.* New York: Marcel Dekker Inc, 1992; 457–484.

ACKNOWLEDGEMENTS. This work was supported by grants from the National Health and Medical Research Council of Australia, and the Women's and Children's Hospital Foundation. We thank Gina Glemente for expert technical assistance.

**Received 4 January 1993; accepted in revised form 1 February 1993**
